# Increased heart rate in high-risk hypertensives related to increased heart failure and sudden death

**Published:** 2010-08

**Authors:** J Aalbers

**Affiliations:** Special Assignments Editor

## Introduction

High-risk hypertensives with a heart rate above 80 beats per minute (bpm) have an increased risk of cardiovascular events, particularly heart failure and sudden death, according to a new analysis of the Valsartan Anti-hypertensive Longterm Use Evaluation (VALUE) trial, presented at the 2010 American Society of Hypertension congress (ASH).^1^ Both baseline and in-trial tachycardia, measured every year by ECG, were strong, independent predictors of cardiovascular events.

The VALUE trial, first reported almost six years ago, showed that valsartan and amlodipine-based therapy were essentially equal in reducing the primary endpoints of cardiac morbidity or mortality in patients with hypertension and at least one high-risk factor.^2^ The study was conducted over five years.

The researchers also identified that most of the risk occurred in those trial participants with heart rates of 79 bpm or more. There was a striking increase in the primary endpoint in the highest quintile of heart rate (≥ 79 bpm) compared with the pooled lower four quintiles.

Annual incidence of new primary endpoint events in the highest quintile (compared with the lower four) was 30% higher in the first year of the study, 55% higher in the third year, 52% more in the fourth, and 46% higher in the fifth year of the study. A similar trend was seen throughout the trial for the heart failure and sudden death components of the endpoint.

The negative effect of the in-trial tachycardia was not modified by the blood pressure control achieved in the study. The relative increase in the primary endpoint of the tachycardic group was 68% in the blood pressure-controlled and 63% in the blood pressure-uncontrolled groups (p < 0.0001).

The results of the SHIfT trial will answer unequivocally whether reducing the heart rate in patients with systolic heart failure with the specific heart ratelowering agent ivabradine, will improve prognosis. The trial randomised to either ivabradine or placebo, heart failure patients in NYHA classes II to IV, with ejection fraction less than 35% and a heart rate greater than 70 bpm, who were already being treated with beta-blockers, ACE inhibitors and diuretics. The primary endpoint of the study is cardiovascular death and hospitalisation for heart failure.

The SHIfT study is the largest ever study undertaken in heart failure, with 6 500 patients. The results of the SHIfT study will be announced at the hotline session on 29 August 2010 at the European Society of Cardiology’s annual congress in Stockholm.

This trial is pivotal to the clinician’s perception of the value of heart rate lowering with ivabradine to reduce cardiovascular morbidity and mortality in these high-risk cardiovascular patients.

## References

[1] Julius S, Palatini P, Kjeldsen S (2010). Tachycardia predicts CV events in the VALUE trial. American Society of Hypertension 2010 scientific meeting..

[2] (2004). VALUE study highlights the need for aggressive blood pressure lowering in high-risk patients.. Cardiovasc J Afr.

[3] (15). New ASCOT analysis: Beta-blockers not beneficial in hypertensives with tachycardia hypertension..

